# Accessing Nature’s diversity through metabolic engineering and synthetic biology

**DOI:** 10.12688/f1000research.7311.1

**Published:** 2016-03-24

**Authors:** Jason R. King, Steven Edgar, Kangjian Qiao, Gregory Stephanopoulos

**Affiliations:** 1 Department of Chemical Engineering, Massachusetts Institute of Technology, Cambridge, MA, USA

**Keywords:** metabolic engineering, synthetic biology, natural product discovery

## Abstract

In this perspective, we highlight recent examples and trends in metabolic engineering and synthetic biology that demonstrate the synthetic potential of enzyme and pathway engineering for natural product discovery. In doing so, we introduce natural paradigms of secondary metabolism whereby simple carbon substrates are combined into complex molecules through “scaffold diversification”, and subsequent “derivatization” of these scaffolds is used to synthesize distinct complex natural products. We provide examples in which modern pathway engineering efforts including combinatorial biosynthesis and biological retrosynthesis can be coupled to directed enzyme evolution and rational enzyme engineering to allow access to the “privileged” chemical space of natural products in industry-proven microbes. Finally, we forecast the potential to produce natural product-like discovery platforms in biological systems that are amenable to single-step discovery, validation, and synthesis for streamlined discovery and production of biologically active agents.

## Introduction

Small molecules play an important role in enhancing our understanding of metabolic control in multistep reaction networks that underlie mechanisms of disease and orchestrate industrial biocatalysts. As such, small molecules account for a large fraction of the new drugs introduced each year, especially those emerging from natural products research. Metabolic probes and drug candidates are born from small molecule libraries that are typically limited in structural *diversity*, a key constraint for the discovery of new bioactive small molecules ^1^.

**Figure 1.**
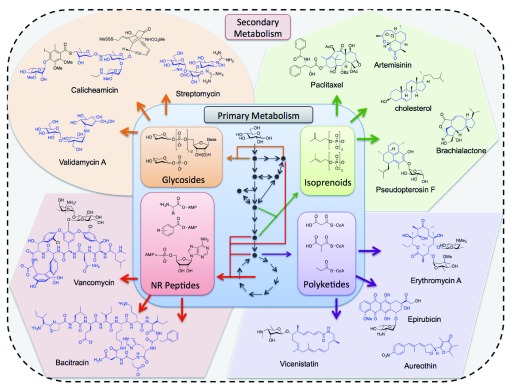
Schematic of natural diversity in secondary metabolism. Complex metabolites diverge from a common pool of primary building blocks. Secondary metabolites and their respective precursors are grouped by colored areas: green (isoprenoids), purple (polyketides), red (non-ribosomal peptides), and orange (glycosides). Paradigmatic structures of each metabolite class are shown with the structure cores highlighted in blue. Colored arrows denote simplified enzymatic transformations. Black arrows and nodes correspond to central metabolism in heterotrophs to denote the origin of primary metabolites from central carbon.

Organic chemists have boundless potential to create drugs with diverse molecular topologies from commodity chemicals using the immense diversity of reactions at their disposal. On the other hand, without selective pressures to guide the chemistry, practical discovery of biologically active agents is limited to the manipulation of known natural compounds and the use of combinatorial high-throughput screens ^2^. The *de novo* synthesis of complex natural products is a cost- and labor-intensive process, requiring world-class expertise. While traditional combinatorial chemistries employed orthogonal reactions to join small, flat, multi-functional building blocks, recent biology-inspired diversity-oriented methodologies are exploring a greater array of chemotypes with increased dimensionality and complexity, as one finds with natural secondary metabolites ( Figure 1) ^1, 2^. Unsurprisingly, chemically derived, biologically active compounds tend to resemble natural products. The similarities inform structural signatures of bioactivity, like the number of stereogenic carbons, scaffold rigidity, and the carbon/heteroatom ratio of the molecules ^2, 3^. Such descriptors of biological activity reveal that natural products provide a pool of “privileged” scaffolds as starting points for molecular probes and drugs ^3^. Combinatorial biosynthesis alleviates many of the concerns with traditional combinatorial chemistry by producing only those compounds with properties similar to natural products. In combinatorial biosynthesis, cells or enzymes are programed for diverse compound generation by systematically switching enzymes in a biosynthetic pathway (e.g. polyketide pathways) or using enzymes with broad substrate ranges (e.g. glycosyltransferases [GTs]) to produce product libraries ( Figure 1) ^4– 6^. Enzyme- and cell-based library generation emulates the natural means for creating chemical diversity by employing genetically encoded catalysts that co-evolve with their products in response to environmental pressures.

Given immense recent interest in natural product biosynthesis and discovery ^2, 7– 12^, here we provide perspective on how synthetic biology and metabolic engineering are enabling compound discovery and biosynthesis. We parameterize natural themes for exploring chemical diversity under the guide of evolution. Finally, we forecast the potential for metabolic engineering to consolidate cell-based platforms for library generation and hit validation, as well as scalable synthesis in the practical discovery of biologically active compounds.

## Engineering small molecule discovery platforms: derivatization *vs.* diversification

**Figure 2.**
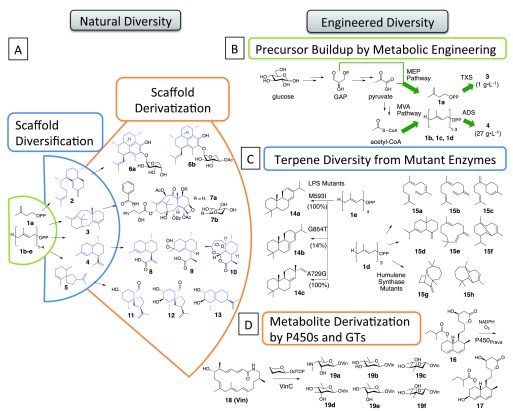
Natural paradigms for compound diversity inspire engineering efforts for compound discovery. **A**) schematic of isoprenoid diversification in which distinct terpenes ( **2–5**) arise from common building blocks ( **1a–e**) and are subsequently functionalized into diverse isoprenoids ( **6–13**); **B**) engineering secondary metabolite production requires augmented flux through biosynthetic pathways to access compound precursors, such as the buildup of isopentenyl diphosphate building blocks for the overproduction of taxadiene ( **3**) ^92^ and amorphadiene ( **4**) ^93^; **C**) scaffold diversification is emulated through enzyme engineering as shown in mutagenesis of the plant-derived levopimaradiene synthase (LPS) ^79^ and humulene synthase ^88^; **D**) scaffold derivatization is performed by engineered enzymes as in the P450-catalyzed hydroxylation of compactin ( **16**) to produce the drug pravastatin ( **17**), or by naturally promiscuous enzymes as with variable glycosylation of vicenilactam ( **18**) with glycosyltransferase VinC ^25, 36^.

Advances in chemical biology and metabolic engineering are providing insights into the biological routes to create natural product diversity while also offering the potential to harness and manipulate this diversity under the guide of selective pressure. Armed with an arsenal of robust genetic tools and proven hosts for prokaryotic (e.g. *Escherichia coli*, *Bacillus subtilis, Streptomyces sp.*) and eukaryotic (e.g. *Saccharomyces cerevisiae*) production platforms, biological engineers have begun exploring diversity in both natural and unnatural contexts ( Figure 2) ^13^. Natural product diversity results from two themes of chemical evolution: *derivatization* of a shared molecular scaffold by variable functionalization of a common core, or *diversification* to enable the synthesis of various scaffold cores with distinct shapes from common building blocks ( Figure 2A). Below we describe recent trends and specific advances that highlight the importance of exploring chemical diversity in molecule discovery and underscore the role of synthetic biology and related fields towards this end.

### Diversity through scaffold derivatization

Chemical transformations of complex molecules often suffer from a lack of regioselectivity and stereoselectivity, poor discrimination between functional groups of similar reactivity, and an incompatibility with biological media. Enzymes, however, catalyze site-specific and stereoselective chemistries in water—often within a microorganism. Numerous enzyme-mediated chemical functionalizations of natural products are known, including scaffold alkylation ^14– 16^, acylation ^17^, oxidation ^18, 19^, glycosylation ^4, 20^, and halogenation ^21^. Here we focus the discussion of enzyme-tailored scaffold derivatization on the mature cases of natural product tailoring by cytochrome P450 oxidases (P450s or CYPs) and GTs. It is worth noting the biosynthetic potential of the lesser-utilized bio-acylation and bio-halogenation reactions for natural product derivatization, as these reactions can introduce orthogonally reactive handles for late-stage library differentiation ^21^.

A robust derivatization strategy employs naturally promiscuous P450s that have been engineered to harness multiple natural and novel chemistries *in vivo* ^22– 24^. For example, Keasling and co-workers used rational enzyme mutagenesis of a plant-mimicking bacterial enzyme (P450-BM3) capable of epoxidizing the plant-derived taxane amorpha-4,11-diene ( Figure 2 [ **4**]) to obtain a more thermostable and selective epoxidation catalyst. P450-BM3 mutant G3A328L enabled the biosynthesis of the value-added compound artemisinic-11 *S*,12-epoxide at 250 mg/L in *E. coli*, thereby improving the semi-synthesis of the antimalarial drug artemisinin ( Figure 2 [ **10**]) ^18^. Recently, McLean *et al.* evolved CYP105AS1 from *Amycolatopsis orientalis* to hydroxylate the pravastatin ( Figure 2 [ **17**]) precursor compactin ( Figure 2 [ **16**]) in the engineered *Penicillium chrysogenum* strain D550662, ultimately achieving titers of 6 g/L of the blockbuster drug after 200 h in a 10 L fed-batch fermentation ( Figure 2D) ^25^.

P450-catalyzed metabolite derivatization is likewise offering avenues to explore chemical space that was previously unavailable in a biological setting. Frances Arnold’s lab has developed an impressive array of P450 catalysts including an engineered diazoester-derived carbene transferase (P450 _BM3_/CYP102A1) for the stereoselective cyclopropanation of styrenes, which have concomitantly become available biologically via the metabolic engineering of *E. coli* for styrene production from L-phenylalanine at a titer of 260 mg/L ^26, 27^. Arnold’s team expanded the work to enable incorporation of the cyclopropane *in vivo* by engineering the electronics of the enzyme active site to accommodate NAD(P)H as an electron donor, and upon altering the active site architecture, they further engineered the catalyst for cyclopropanation of N,N-diethyl-2-phenylacrylamide, a putative intermediate in the formal synthesis of the serotonin and norepinephrine reuptake inhibitor levomilnacipran—marketed by Actavis Inc. as Fetzima for the treatment of clinical depression ^28– 30^. On the chemical front, Wallace and Balskus have developed porphyrin-iron(III) chloride catalysts that function similarly to Arnold’s P450-BM3 mutants while presenting biocompatible reactions with living styrene-producing *E. coli* ^31^. Such approaches highlight the potential to meld chemical and biological approaches for tailored molecule derivatization in engineered organisms ^32, 33^.

GTs are also attracting attention in the derivatization of natural products, including polyketides, non-ribosomal peptides, and terpenoids, for the discovery of novel antimicrobial agents with tailored pharmacological properties, including augmented target recognition and improved bio-availability ^4, 20, 34, 35^. In this regard, dNDP-glycosides ( Figure 1) represent a biosynthetically viable class of saccharide donors for promiscuous and engineered GTs that exhibit substrate tolerances for both the saccharide and aglycone portions of the reaction products. For instance, Minami *et al.* exploited the broad substrate tolerance of vicenisaminyltransferase VinC from *Streptomyces halstedii* HC 34 in the discovery of 22 novel glycosides from 50 sets of reactions for the glycodiversification of natural polyketide scaffolds ( Figure 2D) ^36^. More recently, Pandey *et al.* demonstrated the derivatization of clinically relevant resveratrol glycosides, producing ten different derivatives of the plant-derived metabolite, all accommodated by YjiC, a bacterial GT from *Bacillus licheniformis* ^34, 37^. However, in order for GT-catalyzed glyco-derivatization to be realized *in vivo*, the prerequisite biosynthesis of NDP-glycosides as glycosyl donors and acceptors must be engineered from bacterial monosaccharide and nucleotide triphosphate pools. A key advance in the supply of glycosyl donors was the discovery of the reversibility of GT-catalyzed reactions whereby Thorson and co-workers were able to generate more than 70 analogs of the natural products calicheamicin and vancomycin ( Figure 1) using various nucleotide sugars ^38^. Using OleD as the initial model enzyme, Thorson’s team evolved the substrate tolerance of GTs to enable glycosylation of not only natural products but also non-natural compounds and proteins ^4, 39^. More recently, Gantt *et al.* enabled the rapid, colorimetric screening of engineered GTs, and subsequently evolved an enzyme (OleD Loki) for the combinatorial enzymatic synthesis of 30 distinct NDP-sugars that are putatively amenable to further enzymatic manipulation in common microbial hosts ^4, 40^.

Metabolic engineering strategies for *in vivo* combinatorial glyco-derivatization of secondary metabolites have also demonstrated success by combining heterologous saccharide biosynthesis genes into non-natural pathways. In 1998, Madduri *et al.* demonstrated the fermentation of the antitumor drug epirubicin ( Figure 1) in *Streptomyces peucetius* and sparked intense interest in the role of metabolic engineering and combinatorial biosynthesis for the discovery and production of glyco-pharmaceuticals ^20, 41– 44^. These efforts have begun to impact glyco-engineering in more common microbial hosts, such as an *in vivo* small molecule “glyco-randomization” study in *E. coli* by Thorson and co-workers ^4^ or the variable derivatization of erythromycin by Pfeifer and co-workers ^45^; however, much success for *in vivo* glyco-derivatization remains in *Streptomyces* ^4, 41, 46– 48^.

### Diversity through scaffold diversification

Scaffold derivatization enables the fine-tuning of compound activity by increasing compound resolution in a defined chemical space. The production of novel secondary metabolites through scaffold diversification, on the other hand, is a common theme of biosynthesis in plants and fungi that enables the exploration of completely new areas of chemical space. In order to generate beneficial molecules, it has been proposed that microbes and plants generate a diverse library of small molecules. Many liken these broad ranges of natural products to the host’s chemical “immune system”, where producing compounds with no known target could allow for resistance to an as-yet unencountered pathogen and provide evolutionary fitness of organisms with more diverse natural product portfolios ^49– 51^. Natural metabolite diversification has recently inspired diversity-oriented chemical syntheses that emulate the biological reaction cascades in the generation of new, drug-like scaffolds ^1, 52, 53^. Others have attempted to simplify metabolite archetypes to common core structures that may serve as starting points for discovery through derivatization; however, metabolite profiling of novel compounds from marine life and fungi continues to produce novel scaffold core structures, suggesting that to limit discovery to known scaffolds would severely curb the biosynthetic potential of evolutionary pressure ^2, 54– 56^. Engineering whole cells for scaffold diversification, on the other hand, was recently demonstrated by Wang *et al.*, who combined the biosynthetic potential of plant, fungal, and bacterial enzymes for the production of 12 novel phenylpropanoid derivatives from L-tyrosine and L-phenylalanine in *E. coli* ^57^. Evolva reported the discovery of novel antiviral scaffolds using a heterologous flavonoid biosynthesis platform in *S. cerevisiae* ^3, 58^. By consolidating biosynthesis and screening into a single cell, the team was able to synthesize, validate, and structurally characterize 74 new compounds—28 of which showed activity in a secondary Brome Mosaic Virus assay—in less than nine months ^3^.

Aiding the discovery of new scaffolds, non-ribosomal peptide synthetases (NRPS) and polyketide synthases (PKS) comprise equally useful, and often interconnected, classes of “assembly line” enzymes for *in vivo* scaffold diversification. The utility of NRPS/PKS enzymes for complex scaffold synthesis and elaboration emerges from the simplicity and modularity of their catalytic domains ^59^. Core NRPS/PKS genes encode for ketosynthase (KS), acyl transferase (AT), acyl/peptidyl carrier protein (ACP/PCP), condensation domain (C), and adenyltransferase (A) that catalyze the elongation of the polyketide/ peptide skeleton, and a terminal thioesterase (TE) severs the formed macrolide from the multi-domain synthetase. Along with auxiliary ketoreductase (KR), dehydratase (DH), and enoyl reductase (ER) domains, the core domains allow for the programmable building of variable macrolide and macrolactone scaffolds from divergent pools of ketoacids and amino acids ^17, 59– 61^. Once formed, the core scaffolds are natively derivatized by so-called “tailoring enzymes” to introduce native and non-native functionality as per the discussion of engineered P450s and GTs ( *vide supra*) ^59^.

**Figure 3.**
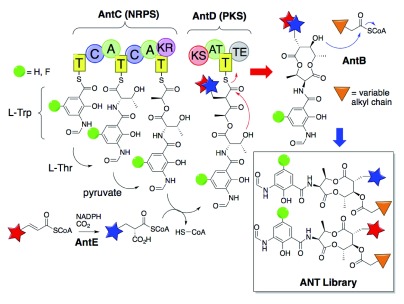
Engineered diversification and derivatization of antimycin (ANT) scaffolds by a promiscuous PKS/NRPS (adapted from Yan *et al.*, 2013) ^17^. Domain key: T = thioylation (e.g. acyl/peptidyl carrier protein ACP/PCP), C = condensation, A = adenylation, KR = ketoreductase, KS = ketosynthase, AT = acyl transacylase, TE = thioesterase. Green ball represents variable use of H or F-modified starting material. Red vs. blue star depicts unsaturated and carboxylated acyl substituent at C7, respectively. Orange triangle depicts variation in the alkyl chain of the C8 acyl substituent.

The modularity of the biosynthetic machinery of NRPS/PKS megasynthases allows for the rational engineering of combinatorial biosyntheses to access novel chemical space ^7, 62^. Combinatorial NRPS/PKS systems have enabled predictable changes to the scaffold core, derived from three programmable inputs into the biosynthesis. The inputs include the following: 1) variable use of organic building blocks such as short-chained acyl-coenzyme A (CoA) molecules or amino acids for the chain elongation step of scaffold synthesis ^61, 63– 68^; 2) chain length variations originating from KS and TE engineering ^69– 70^; and 3) alterations in the reduction program of the scaffold as a result of DH, KR, and ER engineering ^71– 72^. Yan *et al.* exemplified the biosynthetic potential to diversify antimycin (ANT) scaffolds through the metabolic engineering of promiscuous NRPS/PKS enzymes in the ANT-producing *Streptomyces sp.* NRRL 2288 ( Figure 3) ^17^. Following the *in vivo* production of ANT scaffolds with variable fluorination at C5’ and alkylation at C7, the authors further derivatized the ANT library at C8 with a promiscuous acylating protein (AntB) and various acyl-CoAs *in vitro*, generating 380 total and 356 novel ANT variants. Chemler *et al.* recently exploited the biosynthetic prowess of homologous recombination—a natural paradigm of NRPS/PKS evolution—to create PKS libraries for the programmable biosynthesis of engineered polyketide chimeras of known macrolide and macrolactone antibiotics pikromycin and erythromycin ( Figure 1) ^60^. Similarly, Sugimoto *et al.* demonstrated that engineering of an artificial PKS pathway by domain swapping in *Streptomyces albus* allowed reprogramming of the aureothin ( Figure 1) system for production of luteoreticulin and novel derivatives thereof ^73^. Despite significant challenges for NRPS/PKS engineering ^5, 71, 74^, recent successes with homologous recombination and structure-guided domain swapping of NRPS/PKS’s, coupled to the increased efficiency of Cas9-accelerated gene editing, forecast a time when functional NRPS/PKS variation may be routine ^6, 60, 64, 66, 73, 75– 76^.

Isoprenoids, enumerating over 55,000 compounds, comprise perhaps the richest source of diversity among secondary metabolites ^77^. The ability to emulate the natural evolution of this diversity will likely allow the access to known and new plant-derived isoprenoids ( Figure 2). Isoprenoid biosynthesis is characterized by four reactions of the five-carbon units isopentenyl pyrophosphate (IPP) and dimethylallyl pyrophosphate (DMAPP): chain elongation, branching, cyclopropanation, and cyclobutanation ^77^. Metabolic engineers have manipulated microbial pathways around the chain elongation reaction to build up terpenoid precursors of different lengths and stereochemistries, including IPP/DMAPP ( Figure 2 [ **1a–b**]), geranyl diphosphate ( Figure 2 [ **1c**]), farnesyl diphosphate ( Figure 2 [ **1d**]), and others ^78– 83^. The strategy for introducing diversity as well as directing flux to a desired metabolite then comes from the subsequent pathway and enzyme engineering of terpene synthases that cyclize these building blocks, forming various scaffolds amenable to derivatization with downstream enzymes ( Figure 2C). A natural paradigm of terpene synthases and cyclases is the combination of substrate specificity with structural plasticity—a pairing of characteristics that enables rapid evolution of the enzyme for the production of product profiles that meet the environmental demands of the host organism ^84^. In support of this hypothesis, multiple groups have confirmed that through evolution and rational engineering, diterpene synthase activities can be altered to produce multiple, non-native terpenes ( Figure 2C) ^9, 84– 88^. Salmon *et al.* demonstrated that a convergent point mutation from a library of the *Artemisia annua* amorpha-4,11-diene synthase (Y420L) enabled the production of numerous cyclized products without compromising catalytic activity ^89^. Rising *et al.* discovered the serendipitous conversion of a non-natural substrate of tobacco 5-epi-aristolochene synthase, anilinogeranyl diphosphate, to the novel paracyclophane terpene alkaloid 3,7-dimethyl-trans,trans-3,7-aza[9]paracyclophane-diene, which they dubbed “geraniline” ^90^. The finding demonstrates that terpene precursor diversity and bioavailability, in addition to terpene synthase engineering, are key inputs for programmable scaffold diversification. The explicit application of terpene diversification to diversity-oriented molecule discovery is gaining interest, but to realize the full biosynthetic potential of terpenes will likely require more insight into the mechanism of terpene synthases and the directed biosynthesis of terpene precursors ^9, 91^.

## Engineering systems from discovery to production

Secondary metabolites are a treasure trove for the discovery of biologically active compounds, but they are metabolically “expensive”, leading organisms to match production to natural demands of the environment. To meet the demands for human need, microbial cells can be engineered to over-produce complex secondary metabolites—typically plant or fungal in origin—at the expense of host resources including energy storage molecules and biomass. High titers of non-native metabolites are possible via rational pathway engineering as shown in the case of taxadiene ( Figure 2 [ **3**]) and amorpha-4,11-diene ( Figure 2 [ **4**]) syntheses in *E. coli*, which detail that metabolite balance through modular pathways is crucial to high production ( Figure 2A) ^92, 93^. Bypassing regulation also allows increased production of native secondary metabolites, as shown recently by Tan *et al.* with validamycin ( Figure 1) biosynthesis in *Streptomyces* ^94^. The team generated a double deletion mutant ( *S. hygroscopicus* 5008 ∆shbR1/R3) to remove feedback inhibition and increase validamycin titers to 24 g/L and productivities to 9.7 g/L/d, which are the highest capacities yet reported ^94^.

Metabolic engineering can harvest synthetic genes from marine, plant, and fungal systems for the production of a diverse set of known compounds including terpenoids, flavonoids, and alkaloids in industrially useful microbial hosts ^70, 95, 96^. The reconstitution of heterologous pathways in fast-growing microbes is akin to hijacking evolution for efficient and expedient production. To this end, modular pathway reconstruction, or “retrobiosynthesis”, effectively maximizes a cell’s capacity to integrate new biological circuits and appropriate valuable cell resources for high secondary metabolite production ^97– 99^. Retrobiosynthesis allows for the systematic evaluation of complex multi-step pathways by isolating key transformations of a complete pathway into a series of independent modules that can be engineered in parallel ^99^. Leonard *et al.* demonstrated modular pathway engineering for the high-level production of levopimaradiene, a branch-point precursor to pharmaceutically relevant plant-derived ginkgolides ^79^. Upon increasing IPP/DMAPP titers through overexpression of mono-erythritol phosphate pathway enzymes in *E. coli* and separately engineering the geranylgeranyl pyrophosphate synthase/levopimaradiene synthase system for increased selectivity and productivity, the team achieved a 700 mg/L titer in a bench-scale bioreactor. This is one of the first applications whereby metabolic engineering was combined with protein engineering to maximize production and selectivity of a desired compound.

Synthetic consortia offer another tool in which the metabolic burden of complex molecule synthesis can be distributed over multiple hosts. Recently, Zhou *et al.* engineered a cross-kingdom co-culture to produce oxygenated taxane precursors to the potent, plant-derived, anti-tumor drug paclitaxel ( Figure 1), achieving titers of 33 mg/L ^100^. Mimicking the general engineering strategy for spatially controlled production of branched-chain alcohols and mevalonate-derived terpenes in yeast ^101, 102^, Zhou *et al.* combined the divergent advantages of efficient cytochrome P450 expression in *S. cerevisiae* and the efficient taxadiene ( Figure 2 [ **3**]) production in *E. coli* ^92^. The system emulates the native plant platform in which oxygen-sensitive taxadiene production is sequestered from the subsequent oxidations to form paclitaxel and other oxygenated taxanes in the peroxyzome ^100, 102^. The synthetic consortium put forth by Zhou *et al.* could represent a natural paradigm of plant isoprenoid production in plant-associated endophytes, further validating the general premise whereby metabolic engineering allows for the directed use of natural evolution for success in biosynthesis ^103^.

In the post-genomic era, gene mining for compound discovery is adding to the engineer’s toolbox. Hwang and others purport that multiplexed “omics” and bioinformatics enable the simultaneous identification of bacterial biosynthetic gene clusters, their encoded enzymes, and the structures of the resultant secondary metabolites for streamlined discovery of molecular structure and function ^41, 104– 106^. Systems-level analyses will further aid compound discovery by unveiling biosynthetic pathways of unknown secondary metabolites and antibiotics in actinomycetes and other organisms ^107– 109^. Of spectacular interest is the growing evidence for compound discovery by bioprospecting “unculturable” actinomycetes and uncharacterized bacteria by mapping, transforming, and editing their DNA heterologously in genetically tractable hosts with “out-of-the-box” genetic systems and clever metabolic engineering ^110– 114^.

Streamlined molecule discovery and production is likewise aided through the engineering of microbial systems for concomitant compound discovery, validation, and scale-up (e.g. the yeast discovery platform from Evolva; *vide supra*) ^3^. Recently, DeLoache *et al.* engineered *S. cerevisiae* to fluoresce orange in the presence of L-3,4-dihydroxyphenylalanine (L-DOPA), an early intermediate en route to (S)-reticuline, and purple in the presence of L-dopaquinone, an unwanted byproduct of L-DOPA oxidation ^115^. PCR mutagenesis of a tyrosine P450 oxidase (CYP76AD1) produced a yeast library that could be easily screened by comparison of the orange:purple fluorescence of single cells with flow cytometry. The team identified P450 mutant CYP76AD1 ^W13L F309L^ as a selective catalyst for reduced L-DOPA production and continued to engineer a *de novo* pathway for (S)-reticuline production from glucose at titers of 80.6 µg/L. Albeit low titer, this approach has already been recognized for the ability to streamline microbial opioid production ^116^.

## Conclusions

A reinvigoration of the potential for engineered enzymes and microorganisms to explore foreign biochemical space and discover molecular probes and therapeutics is clear from a number of recent commentaries and reviews ^9, 11, 41, 117^. Here, we describe examples from enzyme and pathway engineering to illustrate the successes, promises, and challenges for mining the plant, fungal, and microbial metabolomes to produce natural product-like molecules. We outline the underlying themes whereby nature explores chemical diversity through the diversification and derivatization of secondary metabolites—a robust strategy that has inspired recent diversity-oriented chemical syntheses. The co-evolution of natural products with their biosynthetic enzymes in response to environmental pressures is a theme whereby natural diversity begets evolutionary fitness. Several factors highlight the burgeoning potential of modern metabolic engineering to explore chemical diversity: 1) incredible investment into the genetic characterization of secondary metabolism over the last two decades has led to organization of natural product biosyntheses into standardized data sets ^12, 118^; 2) engineering promiscuous, biosynthetic enzymes has allowed for the DNA-encoded diversification of natural product libraries; 3) successful metabolic engineering of industrially proven microbes has allowed complex metabolite biosyntheses with high titers and productivities, and 4) recent technical advances for efficient homologous recombination and consolidated bioprospecting are allowing for biosynthetic compound library creation, validation, and scale-up with increasing simplicity. Perhaps a most critical advantage is that, once an active new structure is identified, through either scaffold derivatization or diversification in an engineered microbe, an actual biochemical process is also available for the synthesis of the target compound in substantial amounts required for toxicity and clinical trials. This path is more efficient compared to the many steps of a new chemical synthesis approach typically followed when promising compounds are identified from prospecting samples of natural sources. Broadly, the potential to apply metabolic engineering to access chemical diversity inspired by natural product biosynthesis illustrates an elegant pairing of science and engineering for biochemical progress.
